# Clinical characteristics and mixed infection patterns of ocular surface infection with Epstein-Barr virus

**DOI:** 10.1186/s12348-026-00579-w

**Published:** 2026-03-31

**Authors:** Zihan Shen, Yiyun Liu, Yilin Liu, Pei Zhang, Yingyu Li, Zekai Li, Hong Qi

**Affiliations:** 1 Institute of Medical Technology, Peking University Health Science Center, Beijing, China; 2 Department of Ophthalmology, Beijing Key Laboratory of Restoration of Damaged Ocular Nerve, Peking University Third Hospital, Beijing, 100191 China

**Keywords:** Epstein-Barr virus, Ocular surface infection, Ocular surface scrapings, Co-infection, Retrospective study

## Abstract

**Purpose:**

To analyze the clinical features of ocular surface diseases caused by Epstein-Barr virus (EBV) infection.

**Methods:**

A retrospective case series study was conducted. Data from 48 patients (54 eyes) with EBV infection who visited Peking University Third Hospital between January 2023 and October 2025 were collected. Patient demographics and baseline information were recorded. Ophthalmic slit-lamp examination, ocular surface (conjunctiva/cornea) scrapings, bacterial culture of ocular secretions, real-time fluorescence quantitative PCR detection, EBV-specific antibody testing, and metagenomic next-generation sequencing (mNGS) were performed.

**Results:**

Among patients infected with EBV on the ocular surface, the majority were middle-aged individuals in the 31–40 age group. The primary risk factors for onset were keeping pets (10/48), followed by colds (6/48); among those keeping pets, parrots were the most common (5/9). The main clinical manifestations were foreign body sensation (37/54) and yellow discharge (34/54). Common signs included mixed conjunctival hyperemia (31/54), follicles on the lower eyelid conjunctiva (17/54), papillae on the upper eyelid conjunctiva (8/54), and punctate epithelial defects on the cornea (17/54). In most ocular surface scrapings, small round lymphocytes were observed alongside a small number of reactive lymphocytes (44/54), which could simultaneously present with a large number of neutrophils (36/54). There was a significant difference between the presence of yellow discharge and the type of conjunctival hyperemia $({{\rm{\chi }}^{\rm{2}}}{\rm{ = 9}}{\rm{.818,}}\;{\rm{P = 0}}{\rm{.007}})$. However, no statistically significant correlation was found between the presence of yellow discharge and the presence of neutrophils in the scraping results $({{\rm{\chi }}^{\rm{2}}}{\rm{ = 0}}{\rm{.64,}}\;{\rm{P > 0}}{\rm{.05}})$. Significant differences were found in EBV viral loads among different groups of combined symptoms $(\:\mathrm{F=4.207,P=0.02})$ and among different follicle groups $(\:\mathrm{F=4.466,P=}\mathrm{0.007})$. No statistically significant correlation was found between the lymphocyte count in the scraping and the EBV viral load in the affected eye $(\:\mathrm{F=0.49,P>0.05})$.

**Conclusion:**

EBV infection of the ocular surface is prone to concurrent infections; therefore, a detailed medical history inquiry is crucial. Ocular surface tissue scraping examination can rapidly identify viral infection-related inflammatory characteristics and rule out bacterial/fungal infections, providing effective supportive auxiliary diagnostic evidence for viral ocular surface infection, and precise diagnosis of EBV infection needs to be achieved in combination with molecular biological and serological tests.

Epstein-Barr virus (EBV), a member of the γ-herpesvirus subfamily, is classified as human herpesvirus type 4 (HHV-4). It is characterized by an extremely high global prevalence; approximately 90% of adults are seropositive, having been infected during childhood. Primary infection often leads to infectious mononucleosis (IM), clinically presenting with fever, pharyngitis, and lymphadenopathy. EBV is primarily transmitted via saliva and possesses the ability to infect B lymphocytes and epithelial cells, establishing a lifelong latent infection within the host [1]. In most cases, this latent infection is either asymptomatic or presents with only mild symptoms; following infection, the virus can establish a prolonged, lifelong latency within the host [2], however, when the host’s immune function is compromised or under specific pathological conditions, the latently infected virus can be reactivated, thereby initiating a cascade of complex pathophysiological processes [3]. The pathogenic spectrum of Epstein-Barr virus (EBV) is remarkably broad. While it is closely associated with infectious mononucleosis, it has been firmly established as a causative agent for various malignancies, including lymphomas, Kaposi’s sarcoma, and ocular tumors [4].

In the field of ophthalmology, the potential threat posed by EBV cannot be overlooked. Accumulating evidence suggests that EBV is closely associated with a diverse spectrum of ocular diseases, ranging from superficial infections of the ocular surface to deep intraocular involvement, and even extending to the optic nerve, such as eyelid-facial inflammation syndrome, retinitis, optic nerve papillitis, uveitis, keratitis, and pterygium [5–10].In the context of ocular surface infections, although EBV is not the most prevalent pathogen, it has been identified as a significant viral etiology for acute conjunctivitis in specific geographic regions, such as Vanuatu, where it ranks second only to adenovirus [11]. In existing literature, EBV-related conjunctivitis is frequently reported as follicular conjunctivitis [10]. When EBV involves the cornea, it primarily manifests in three distinct forms: (1) Subepithelial Infiltrative Type: Clinically resembling Thygeson superficial punctate keratitis, characterized by recurrent episodes of superficial punctate opacities; (2) Bilateral Annular Interstitial Keratitis: Commonly observed in young patients with concurrent systemic infectious mononucleosis; and (3) Multifocal Non-suppurative Keratitis: Involving the full thickness or deep layers of the peripheral cornea, which may subsequently lead to corneal neovascularization [7, 12]. Studies have also demonstrated that EBV-related keratitis most frequently presents as the epithelial or stromal type. The epithelial form may be accompanied by punctate epithelial keratitis, dendritic keratitis, or geographic keratitis; whereas the stromal type may exhibit multifocal infiltrates in the anterior to mid-stroma (resembling adenoviral keratitis), active inflammatory plaques, or diffuse infiltrates, or deep peripheral infiltrates with or without vascularization (resembling syphilitic interstitial keratitis) [13, 14].

Despite the growing recognition of its association with ocular diseases, the diagnosis and management of EBV-related ocular surface infections remain clinically challenging. Clinically, these infections often present with non-specific symptoms that can easily be confused with those caused by other common pathogens such as adenovirus or herpes simplex virus.Furthermore, current diagnostic strategies primarily rely on a combined approach of clinical evaluation and laboratory testing. While polymerase chain reaction (PCR) assays and serological detection of specific antibodies are considered the gold standard, they are often limited by the time required for results—typically taking 2–3 days—which frequently exceeds the optimal therapeutic window for intervention [2, 7, 10, 15]. While traditional rapid smear microscopy can efficiently exclude bacterial or fungal infections, it lacks the specificity required to identify viral pathogens such as EBV, thereby limiting its utility in establishing a definitive diagnosis for viral ocular surface diseases [16], however, smear microscopy cannot directly visualize the typical morphology of EBV and is therefore not a core diagnostic method for EBV infection. This diagnostic lag and limitation often force clinicians to rely heavily on personal experience for empirical treatment during the early stages of illness, resulting in significant uncertainty regarding the diagnosis and management of EBV-related ocular surface infections.

At present, research on EBV-infected ocular surface diseases lacks representative clinical feature analysis. Diagnosis is easily confused with other pathogens, and the testing process is time-consuming. Consequently, patients often receive empirical treatment based on clinicians’ personal experience during the early stages of illness, making the diagnosis and treatment of EBV-infected ocular surface diseases extremely difficult. Therefore, the medical community urgently needs representative clinical feature analysis to clarify the true nature of EBV ocular infections. In view of this, our study conducted a retrospective analysis of patients diagnosed with EBV viral infection at Peking University Third Hospital from January 2023 to October 2025. We systematically explored the epidemiological characteristics, clinical manifestations, laboratory test results, and their internal associations of EBV-infected ocular surface diseases. We aim to provide ophthalmologists with clearer and more practical evidence-based medical evidence to enhance early recognition capabilities and improve the level of precise diagnosis and treatment.

## Materials and methods

### Study population

The study was designed as a single-center, retrospective observational analysis. It has been approved by the Ethics Committee of Peking University Third Hospital, which waived the requirement for informed consent (Approval Number: M20250430). Based on the inclusion criteria outlined in Sect.  1, we collected data from patients diagnosed with EBV viral infection who visited our hospital between January 2023 and October 2025.

#### Definition and diagnostic criteria

EBV ocular surface infection: Refers to ocular surface inflammatory lesions with positive EBV-DNA detection in local ocular surface samples (conjunctival sac secretions/ocular surface scrapings) by real-time fluorescent quantitative PCR (qPCR), combined with typical ocular irritation symptoms and conjunctival hyperemia signs observed under slit-lamp microscopy. According to serological test results and clinical manifestations, it is further divided into primary EBV ocular surface infection (positive for EBV-specific IgM antibodies, no history of EBV infection, accompanied by systemic acute infection manifestations) and EBV reactivation-related ocular surface inflammation (negative for EBV-specific IgM antibodies, positive for IgG antibodies, with latent EBV infection in the body and ocular surface lesions induced by immune dysfunction or other triggering factors).

The diagnostic criteria for the study subjects were strictly formulated as follows: (1) Presence of typical ocular irritation symptoms (foreign body sensation, increased discharge, blurred vision, epiphora, etc.); (2) Conjunctival hyperemia (ciliary congestion, perilimbal congestion or mixed congestion) was observed in the affected eye under slit-lamp microscopy; (3) Positive EBV-DNA detection by real-time fluorescent quantitative PCR in local ocular surface samples (trace filter paper method/irrigation fluid method); (4) Exclusion of other ocular surface diseases with similar manifestations (such as allergic conjunctivitis, bacterial keratoconjunctivitis, and other viral keratoconjunctivitis without EBV co-infection).

#### Data collection

Data were retrieved from the Outpatient Medical Record System of Peking University Third Hospital. Detailed clinical data of each participant were collected, including basic demographic information (such as gender and age), general characteristics of the illness (etiology, onset time, systemic comorbidities, symptoms, and signs), and auxiliary examination results (slit-lamp biomicroscopy, ocular surface scrapings, bacterial culture of ocular secretions, EBV-specific antibody testing, real-time fluorescent quantitative PCR [qPCR] testing, and metagenomic next-generation sequencing [mNGS]).

##### General information

General information primarily included the patient’s general condition at the time of visit, chief complaint, history of present illness, and past medical history.

##### Clinical signs

Clinical signs were observed under slit-lamp biomicroscopy. Features such as conjunctival hyperemia and edema, follicles on the palpebral conjunctiva, papillae on the palpebral conjunctiva, epithelial defects on the cornea, and corneal epithelial infiltration were recorded. Comparisons were also made with the fellow eye, and detailed records were documented in the medical chart.

#### Ocular ancillary examinations

All patients underwent anterior segment photography using the same manufacturer’s equipment. Prior to examination, a sterile 1% sodium fluorescein solution was instilled into the eye. The light band of the anterior segment camera was adjusted to cobalt blue light for observation and photography. Under normal circumstances, the cornea does not stain; however, epithelial defects appear green, thereby allowing for the assessment of corneal epithelial defects.

#### Virology and laboratory analysis

##### Ocular surface scrapings

A sterile curette was used by an experienced ophthalmologist to collect samples from the affected ocular surface under surface anesthesia. After scraping the lesion area, the collected material was evenly spread as a thin layer on the central portion of a glass slide in one direction with gentle movements. Subsequently, antibiotic eye drops were instilled into the patient’s eye. All prepared smears were sent to the pathology department for Giemsa staining, and cellular features were observed under a microscope.

In this study, the quantity of lymphocytes in ocular surface scrapings was evaluated by a qualitative and semi-quantitative method: experienced ophthalmic pathologists observed Giemsa-stained smears under a microscope, and classified lymphocytes into two grades (“a large quantity of lymphocytes” and “a small number of lymphocytes”) based on the proportion and distribution density of lymphocytes in the visual field. A standardized grading system for lymphocytes was not adopted, and no interobserver consistency validation was conducted in this study.

##### Bacterial culture of ocular secretions

Under aseptic conditions, ocular surface swabs were collected using sterile cotton swabs and inoculated onto blood agar and chocolate agar plates. The plates were incubated at 37 °C for 24 to 48 h. Positive isolates were subjected to mass spectrometry identification using the Zhongyuan Huoji (Zhongyuan Bio) system.

##### qPCR

To obtain ocular surface samples, two distinct collection methods were employed based on clinical feasibility and sample volume requirements. RT-qPCR was uniformly performed for the detection of cytomegalovirus (CMV), Epstein-Barr virus (EBV), adenovirus (ADV), varicella-zoster virus (VZV), herpes simplex virus type 1 (HSV-1) and type 2 (HSV-2) in this study.

- Trace Filter Paper Method: After administering adequate topical anesthesia, a filter paper disc was applied to the target area. The patient was instructed to gently close their eye. Once the disc was fully saturated with tears, it was retrieved using fine-tipped forceps and placed into a sterile centrifuge tube. The tube was immediately capped and sent to the laboratory for analysis.
- Ocular Surface Irrigation Method: A sterile syringe was used to aspirate 0.1 mL of sterile normal saline. The patient was directed to gaze upward, and the saline was instilled into the conjunctival sac. Following a blink reflex, the instilled fluid was aspirated from the ocular surface and transferred into a sterile centrifuge tube. The tube was then capped and submitted to the testing platform for subsequent processing.

In this study, systematic detection of human herpesvirus type 6 (HHV-6), type 7 (HHV-7) and type 8 (HHV-8) was not performed via real-time fluorescence quantitative PCR (qPCR) or metagenomic next-generation sequencing (mNGS). The main reasons for not conducting the above detection were as follows: ① Technically, the qPCR detection platform matched with this study only completed the methodological verification and clinical implementation of detection kits for common ocular surface infectious viruses such as EBV, adenovirus (ADV) and herpes simplex virus (HSV), and there was no standardized detection system for HHV-6/7/8. ② In terms of cost and samples, this was a single-center retrospective study. Restricted by research funding, it was impossible to add the above viral detection items for all samples, and the volume of some samples was limited, which could not meet the sample size requirement for parallel detection of multiple viruses.

##### mNGS

Metagenomic next-generation sequencing (mNGS) is a high-throughput sequencing technology that directly sequences all genetic material in a sample without prior microbial culture, enabling comprehensive detection of all microorganisms (viruses, bacteria, fungi, and other pathogens) in the sample.

A sterile syringe was used to aspirate 0.1 mL of sterile normal saline. The patient was directed to gaze upward, and the saline was instilled into the conjunctival sac. Following a blink reflex, the instilled fluid was aspirated from the ocular surface and transferred into a sterile centrifuge tube. The tube was then capped and submitted to the testing platform for subsequent processing.

#### Serological examination

The decision to perform EBV-specific antibody testing was based on the severity of the patient’s condition and their financial status.

### Statistical analysis

All statistical analyses were performed using SPSS version 26.0 (IBM Corp., Armonk, NY, USA). Quantitative data were expressed as the mean ± standard deviation, whereas categorical data were presented as counts or percentages. For comparisons between groups, an independent-samples t-test was employed for continuous variables, and the chi-square test was used for single-variable categorical data. A P-value of less than 0.05 was considered statistically significant.

Before performing one-way ANOVA, the quantitative data in this study were subjected to the Shapiro-Wilk test for normality and the Levene test for homogeneity of variance, which met the applicable conditions of ANOVA. This study is an exploratory retrospective case study focusing on the correlation analysis of core clinical indicators of EBV ocular surface infection. Multiple comparisons were pre-set research analysis contents and all were directional tests; therefore, Bonferroni or FDR correction was not applied to avoid false negative results caused by over-correction.

## Results

This study retrospectively analyzed patients diagnosed with EBV viral infection at Peking University Third Hospital from January 2023 to October 2025. A total of 48 patients (54 eyes) were included, all of whom presented with ocular hyperemia and tested positive for EBV via ocular surface real-time fluorescence quantitative PCR (qPCR).Through systematic review and analysis of these cases, we explored the following dimensions to clarify the true nature of EBV ocular infections.

### Demographic characteristics

#### Basic characteristics

A total of 48 patients (54 eyes) were enrolled in this study from January 2023 to October 2025, all meeting the inclusion criteria. Among these 48 patients, there were 24 males (24/48) and 24 females (24/48), resulting in a male-to-female ratio of 1:1. In terms of affected eyes, 32 cases were in the right eye (32/54) and 22 cases were in the left eye (22/54).

#### Age distribution

The age range of the 48 patients ranged from 11 to 84 years, with a mean age of 40.5 years. The highest frequency was observed in the 31–40-year-old group (17/48), followed by the 41–50-year-old group (11/48).

#### Seasonal distribution

The seasonal distribution of the 48 patients showed that summer was the peak season for onset, accounting for 22 cases (22/48), followed by autumn (15/48), winter (7/48), and spring (4/48).

#### Onset duration

Regarding the duration of onset in the 54 affected eyes, most patients presented with symptoms within 1–7 days (21/54). A total of 19 eyes (19/54) had an onset duration of 7–30 days, while 14 eyes (14/54) exhibited symptoms lasting more than 30 days.

#### Risk factors

The primary risk factor for EBV infection identified in this study was pet keeping (10/48), followed by common colds (6/48). Among those who kept pets, parrots were the most frequently reported species (5/10). As shown in Table 1.

**Table 1:** Risk factors for ocular surface infection with Epstein-Barr virus (EBV) (*n* = 48 patients)

Predisposing Factor	Number of Cases	Sub-categories (Number of Cases)
No Identifiable Cause	17	
Pet Ownership	10	Parrot (5), Cat (2), Dog (2), Chinchilla (1)
Common Cold	6	
Business Travel	2	
Swimming	2	
Foreign Body in Eye	2	
Allergic Rhinitis	2	
Other Factors	7	Each factor reported in 1 case
Total	48	

Note: The total number of patients is 48, and the number of cases in each sub-category is the number of patients with the corresponding risk factor

### Clinical symptoms and signs

Clinical symptoms were assessed based on patient history, while signs were observed using a slit-lamp biomicroscope.

#### Symptoms

Among the 48 patients, one case (1/48) presented with preauricular lymphadenopathy, and another case (1/48) exhibited retroauricular lymphadenopathy. Regarding ocular symptoms in the 54 affected eyes, foreign body sensation was the most frequently reported complaint, occurring in 37 eyes (37/54). Yellow discharge was present in 34 eyes (34/54), followed by blurred vision in 20 eyes (20/54) and epiphora in 16 eyes (16/54).

#### Signs

Upon slit-lamp examination, conjunctival hyperemia was the most common finding observed in all 54 eyes (54/54). Specifically, 16 eyes (16/54) showed ciliary congestion, 7 eyes (7/54) exhibited perilimbal congestion, and 31 eyes (31/54) demonstrated mixed congestion. Papillary hypertrophy was noted in 8 eyes (8/54), all located on the upper tarsal conjunctiva.

Follicular hyperplasia was identified in 25 eyes (25/54), distributed as follows: 4 eyes (4/25) involved the upper tarsal conjunctiva, 17 eyes (17/25) involved the lower tarsal conjunctiva, and 4 eyes (4/25) had involvement of both upper and lower tarsal conjunctiva.

Other specific signs included chemosis in 1 eye (1/54), salmon patch in 1 eye (1/54), punctate corneal epithelial staining in 17 eyes (17/54), dendritic epithelial keratopathy in 3 eyes (3/54), subepithelial infiltration in 3 eyes (3/54), and corneal ulceration in 2 eyes (2/54).

The distribution of related symptoms and signs is summarized in Table 2.

**Table 2:** Clinical manifestations and signs of the affected eyes in patients with EBV-associated ocular surface diseases (*n* = 54 eyes)

Category	Symptom/Sign	Number of eyes (*n* = 54)
Symptoms	Foreign body sensation	37
	Yellowish discharge	34
	Blurred vision	20
	Epiphora	16
Signs	Bulbar conjunctival congestion	44
	Mixed hyperemia	31
	Palpebral conjunctival follicles	25
	Punctate corneal epithelial staining	17
	Corneal ulcer	2

Note: All signs were observed by slit-lamp biomicroscopy; the number of eyes is the total affected eyes of 48 patients

### Laboratory examinations

#### Ocular surface scrapings

**Fig. 1:**
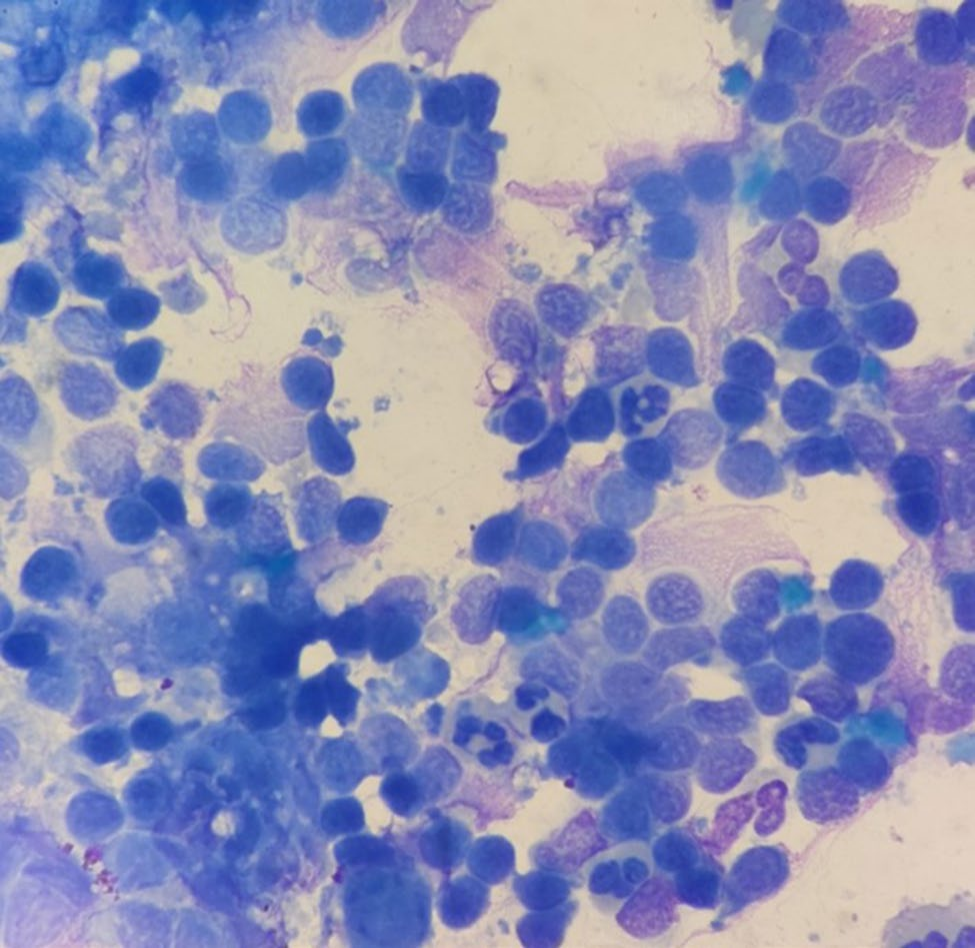
Giemsa staining showing a large number of small round lymphocytes with a few reactive lymphocytes (×1000)

A total of 54 ocular surface scrapings were collected and analyzed using Giemsa staining. In 44 eyes (44/54), inflammatory infiltration dominated by small round lymphocytes with a few reactive lymphocytes was observed (Fig. 1), which is a common non-specific change in viral ocular surface infections and not specific to EBV infection. Among these 44 eyes, 32 (32/44) exhibited a large quantity of lymphocytes, while 12 (12/44) showed only a small number.

Neutrophils were identified in 36 eyes (36/54), with 34 of them co-existing with lymphocytes. Eosinophils were noted in 5 eyes (5/54), of which 4 eyes (4/5) were found alongside lymphocytes. Bacterial cells were detected in 2 eyes (2/54), co-occurring with neutrophils.

**Fig. 2:**
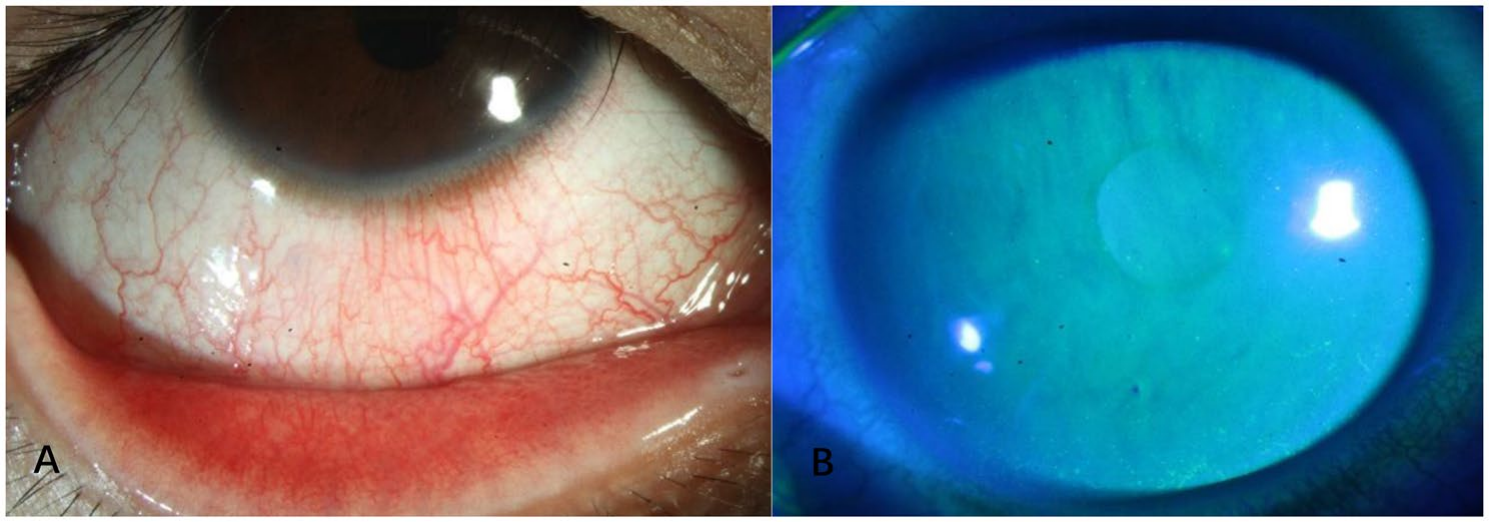
Pure Epstein-Barr virus (EBV) infection: Anterior segment photograph and fluorescein staining photograph of the affected eye (×40 magnification). (**A**) Anterior segment photograph showing the typical ocular surface signs of pure EBV infection, including diffuse mixed conjunctival hyperemia (ciliary and bulbar conjunctiva co-involved), without obvious corneal opacity or subepithelial infiltration. (**B**) Fluorescein staining photograph under cobalt blue light showing mild punctate epithelial defects of the central and paracentral cornea (presenting as scattered green-stained foci), consistent with the punctate epithelial keratopathy of EBV ocular surface infection; no dendritic lesions, geographic epithelial defects or corneal ulceration were observed, and the corneal limbus showed no obvious neovascularization. Note: The case was a 29-year-old male with a history of chinchilla feeding (a risk factor for EBV ocular surface infection in this study), presenting with typical foreign body sensation, yellowish discharge and blurred vision; ocular surface qPCR detected positive EBV-DNA (viral load: 1.05 × 10³ copies/mL), and EBV-specific antibody testing showed negative IgM and positive IgG, confirming latent EBV reactivation

The typical anterior segment manifestations and fluorescein staining characteristics of pure EBV ocular surface infection, corresponding to the above cytological changes, are presented in Fig. 2.

#### Bacterial culture of ocular secretions

Bacterial culture was performed on ocular secretions collected from all 54 affected eyes. A total of 4 cases (4/54) yielded positive results. Specifically, two isolates were identified as Staphylococcus epidermidis and two isolates were identified as Staphylococcus aureus.

#### qPCR

qPCR was performed on ocular samples to determine the viral load of EBV. The viral load ranged from 9.28 × 10¹ to 1.5 × 10⁴ copies/mL, with a mean value of 2.7 (± 3.4) × 10³ copies/mL. This range of EBV viral load was positively correlated with the severity of clinical symptoms of EBV ocular surface infection; that is, the higher the viral load, the more prominent the clinical symptoms such as foreign body sensation and increased discharge in the affected eyes, and samples with high viral load were more likely to be accompanied by ocular signs such as punctate corneal epithelial defects.

Regarding co-infections, 11 eyes (11/54) were found to be positive for other viral pathogens. Specifically, adenovirus (ADV) was detected in 9 cases (9/54), while herpes simplex virus (HSV) was identified in 2 cases (2/54). Notable differences in corneal findings, clinical symptoms and therapeutic management were observed between ADV-EBV and HSV-EBV co-infection cases. For ADV-EBV co-infections, the main corneal findings were superficial punctate keratitis (SPKs) (8/9 eyes) with no dendritic lesions, accompanied by severe mixed conjunctival hyperemia and increased yellow discharge; all these cases were treated with the same oral acyclovir combined with topical antiviral eye drops (adenovirus-specific) for 1–2 weeks. For HSV-EBV co-infections, both cases presented with corneal dendritic lesions and SPKs, associated with severe ocular pain and photophobia (more prominent than single EBV infection); these two cases were given an extended course of oral acyclovir (2 weeks) plus topical ganciclovir gel (qid) with close follow-up, and topical corticosteroids were avoided to prevent HSV reactivation. Five patients had bilateral ADV positivity with unilateral EBV positivity, and all of these patients presented with clinical symptoms in both eyes; for the remaining patients with unilateral EBV positivity, some also had clinical symptoms in the fellow eye without detectable EBV or other co-infecting pathogens.

**Fig. 3:**
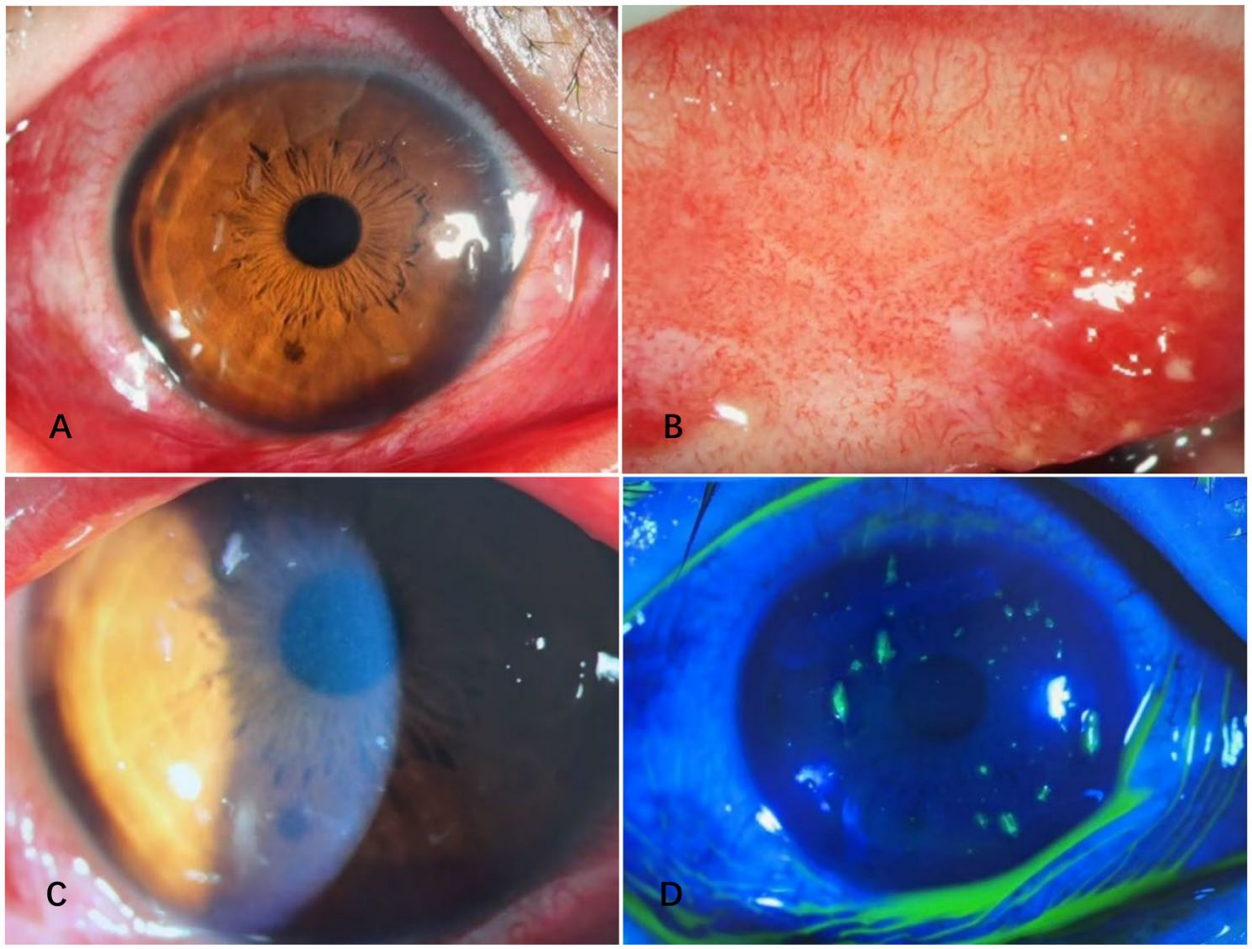
Epstein-Barr virus (EBV) co-infected with adenovirus (ADV): Anterior segment photograph and fluorescein staining photograph of the affected eye (×40 magnification). (**A**) Anterior segment photograph showing severe diffuse mixed conjunctival hyperemia involving the ciliary, perilimbal and bulbar conjunctiva; no conjunctival follicular hyperplasia, ocular discharge, chemosis or neovascularization was observed in the ocular surface. (**B**) Anterior segment photograph of the upper tarsal conjunctiva displaying obvious follicular hyperplasia with round, translucent, well-demarcated follicles distributed densely, a typical viral infection-related conjunctival sign in EBV-ADV co-infection. (**C**) Slit-lamp photograph showing corneal epithelial infiltration in the paracentral area with faint grayish-white opacities; the infiltration foci are scattered, non-ulcerative, and without obvious epithelial erosion, a characteristic sign of ADV infection. (**D**) Fluorescein staining photograph under cobalt blue light showing punctate and patchy green staining in the corneal epithelial layer, corresponding to the corneal epithelial damage induced by combined EBV and ADV infection; no dendritic lesions or geographic epithelial defects were observed. Note: The case was a 39-year-old male with the affected right eye and a history of unclean contact; the patient presented with typical foreign body sensation. Both eyes were positive for ADV-DNA by ocular surface qPCR, while the right eye was positive for EBV-DNA with a viral load of 1.68 × 10² copies/mL. EBV-specific antibody testing showed negative IgM and positive IgG, confirming latent EBV reactivation in the right eye combined with acute bilateral ADV infection

The typical anterior segment manifestations and fluorescein staining characteristics of EBV co-infected with ADV, corresponding to the above molecular detection results, are presented in Fig. 3.

#### mNGS

**Fig. 4:**
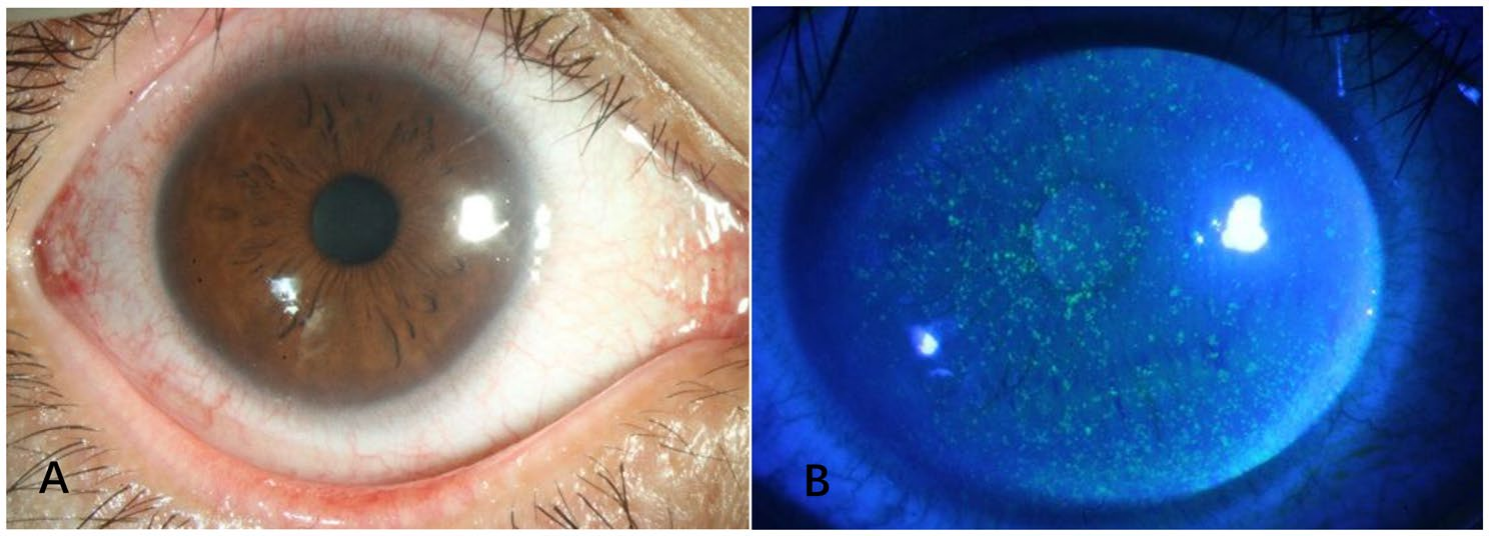
Epstein-Barr virus (EBV) co-infected with Encephalitozoon hellem: Anterior segment and fluorescein staining photographs of the affected eye (×40 magnification) (**A**) Anterior segment photograph showing mild mixed conjunctival hyperemia involving the bulbar and perilimbal conjunctiva; no ocular discharge, chemosis, or neovascularization was observed on the ocular surface. (**B**) Fluorescein staining photograph under cobalt blue light showing diffuse punctate green staining in the corneal epithelial layer; no extensive epithelial defects, dendritic lesions, or geographic staining were detected. Note: The case was a 35-year-old male with a history of parrot feeding and recent cold, presenting with typical foreign body sensation; ocular surface qPCR detected positive EBV-DNA with a viral load of 1.2 × 10⁴ copies/mL, and metagenomic next-generation sequencing (mNGS) of ocular surface irrigation fluid confirmed the presence of Encephalitozoon hellem; EBV-specific antibody testing showed negative IgM and positive IgG, confirming latent EBV reactivation combined with Encephalitozoon hellem infection

Of the 48 patients, 2 underwent monocular mNGS testing, and both were positive for Encephalitozoon hellem. The characteristic anterior segment manifestations and corneal fluorescein staining features of EBV co-infected with Encephalitozoon hellem are presented in Fig. 4.

#### EBV-specific antibodytesting

Serum samples from 30 patients were analyzed for EBV-specific antibodies to distinguish primary infection from latent virus reactivation. The results showed that all 30 tested samples were negative for EBV early antigen IgM and viral capsid antigen IgM, while 29 samples were positive for viral capsid antigen IgG and nuclear antigen IgG. The complete absence of EBV-specific IgM antibodies and the high positive rate of IgG antibodies indicated that none of the enrolled patients had acute primary EBV infection, and the ocular surface lesions in these cases were all associated with the reactivation of latent EBV in the body. No direct laboratory tests (e.g., lymphocyte subsets, immunoglobulin levels) were performed to assess the patients’ immune status in this study. Immune dysfunction was only inferred indirectly based on three aspects: the reactivation of latent EBV indicated by antibody profiles, the identified immune-related risk factors for onset (e.g., colds, autoimmune diseases, pet contact), and clinical manifestations including a high rate of polymicrobial co-infection and asymmetric ocular symptoms in the fellow eye.

### Further analysis

#### Association between symptoms and signs

A chi-square $({{\rm{\chi }}^{\rm{2}}})$ test was conducted to analyze the association between symptoms and types of conjunctival hyperemia. The results indicated a significant difference regarding the presence of yellow discharge $({{\rm{\chi }}^{\rm{2}}}\mathrm{=}\mathrm{9.818,P=0.007})$. No significant association was found between foreign body sensation and the type of conjunctival hyperemia $(\:\mathrm{P=0.}\mathrm{146})$; the associations of blurred vision $(\:\mathrm{P=0.07})$ and epiphora $(\:\mathrm{P=0.}\mathrm{101})$ with the type of conjunctival hyperemia were not statistically significant, but a potential trend of association was suggested. Further analysis revealed that among patients with EBV-associated ocular surface disease, eyes presenting with yellow discharge primarily exhibited mixed congestion, whereas eyes without yellow discharge were predominantly characterized by simple conjunctival hyperemia. See Table 3 for details.

**Table 3:** Association between ocular symptoms and types of conjunctival hyperemia in EBV-associated ocular surface infection (*n* = 54 eyes)

Symptom	Ciliary hyperemia	Perilimbal hyperemia	Mixed hyperemia	Total	x^2^	*P* value
Yellow discharge	3	6	25	34	9.818	**0.007**
Foreign body sensation	4	14	19	37	3.843	0.146
Blurred vision	0	8	12	20	5.308	0.07
Epiphora	4	6	6	16	4.586	0.101

Note: Statistical analysis was performed using the chi-square (χ²) test; *P* < 0.05 was considered statistically significant

#### Association between symptoms and ocular scraping findings

A chi-square test was conducted to analyze the correlation between symptoms and ocular scraping findings. The results showed no statistically significant correlation regarding the presence of yellow discharge and the detection of neutrophils in the scraping samples.

$({{\rm{\chi }}^{\rm{2}}}{\rm{ = 0}}{\rm{.635,}}\;{\rm{P > 0}}{\rm{.05}})$. See Table 4 for details.

**Table 4:** Association between yellow discharge and neutrophil infiltration in ocular surface scrapings (*n* = 54 eyes)

Group	With neutrophil infiltration	Without neutrophil infiltration	Total
With Yellow Discharge	24	10	34
Without Yellow Discharge	12	8	20
Total	36	18	54
x^2^	0.635		
P	0.425		

Note: Statistical analysis was performed using the chi-square (χ²) test; *P* > 0.05 was considered statistically significant, with no significant correlation between the two variables

#### Association between EBV viral load and other clinical outcomes

##### Symptoms

Using the multisymptom counting method, the eyes were categorized into three groups based on the cumulative number of symptoms present: Group 1 (no symptoms), Group 2 (1–3 symptoms), and Group 3 (4 symptoms). Combined with the distribution characteristics of EBV viral load in this study, a one-way ANOVA test revealed significant differences in EBV viral load among these groups $(\:\mathrm{F}\mathrm{=4.207,}\:\mathrm{P=0.0}\mathrm{2})$, which further verified the positive correlation between viral load and symptom severity. LSD post-hoc tests indicated that significant differences existed between Group 1 and Group 2, as well as between Group 1 and Group 3; however, no significant difference was found between Group 2 and Group 3. See Tables 5 and 6 for details.

**Table 5:** One-Way ANOVA of EBV-DNA loads among different concurrent symptom groups (*n* = 54 eyes, ×10³ copies/mL)

	Sum of Squares	df (Degrees of freedom)	Mean Square (MS)	F	*P*
Between Groups	88.191	2	44.096	4.207	0.020
Within Groups	534.535	51	10.481		
Total	622.726	53			

Note: Statistical analysis was performed using one-way analysis of variance (ANOVA); the data met the normal distribution and homogeneity of variance (Shapiro-Wilk test *P* > 0.05, Levene test *P* > 0.05); *P* < 0.05 was considered statistically significant

**Table 6:** LSD post-hoc pairwise comparison of EBV-DNA loads among different concurrent symptom groups (*n* = 54 eyes, ×10³ copies/mL)

Comparison (I-J)	Mean Difference (I-J) (×10³ copies/mL)	Standard error (SE)	*P* value
Group 1 vs. Group 2	5.252	1.932	0.009
Group 1 vs. Group 3	6.244	2.234	0.007
Group 2 vs. Group 3	0.992	1.317	0.455

Note: Group 1 (no symptoms), Group 2 (1–3 symptoms), Group 3 (4 symptoms); statistical analysis was performed using LSD post-hoc test after one-way ANOVA; *P* < 0.05 was considered statistically significant

##### Signs

Based on the presence of follicles in the conjunctiva, the eyes were categorized into four groups: Group 1 (no follicles), Group 2 (only upper eyelid), Group 3 (only lower eyelid), and Group 4 (both upper and lower eyelids). A one-way ANOVA test revealed significant differences in EBV viral load among these groups($\:\mathrm{F}\mathrm{=4.466}\mathrm{,P=0.0}\mathrm{07}$). LSD post-hoc tests further indicated that significant differences existed between Group 1 and Group 2, as well as between Group 2 and Group 3, and between Group 2 and Group 4; however, no significant differences were found between Group 1 and Group 3, Group 1 and Group 4, or Group 3 and Group 4. See Table 7 for details.

**Table 7:** EBV-DNA loads and LSD post-hoc comparison among different conjunctival follicle groups (*n* = 54 eyes, ×10³ copies/mL)

Group	Mean EBV-DNA load ± SD (×10³ copies/mL)	*P* value (vs. Group 1, LSD)	*P* value (vs. Group 2, LSD)	*P* value (vs. Group 3, LSD)	*P* value (vs. Group 4, LSD)
Group 1 (No follicle)	2.86(±3.4)		0.005		
Group 2 (Upper eyelid)	7.81(±5.84)	0.005		<0.001	0.005
Group 3 (Lower eyelid)	1.67(±1.99)		<0.001		
Group 4 (Both eyelids)	1.25(±0.58)		0.005		

Note: Statistical analysis was performed using one-way ANOVA followed by LSD post-hoc test; the data met the normal distribution and homogeneity of variance; *P* < 0.05 was considered statistically significant

##### Ocular Scraping Findings

A one-way ANOVA test revealed no statistically significant correlation between the lymphocyte count in ocular scrapings and the EBV viral load $(\:\mathrm{F=0.49,}\:\mathrm{P}\mathrm{>0.05})$. See Table 8 for details.

**Table 8:** One-Way ANOVA of EBV-DNA loads among different lymphocyte count groups in conjunctival scraping (*n* = 54 eyes, ×10³ copies/mL)

	Sum of Squares	df (Degrees of freedom)	Mean Square (MS)	F value	*P value*
Between Groups	11.741	2	5.871	0.49	0.615
Within Groups	610.985	51	11.98		
Total	622.726	53			

Note: Lymphocyte count was divided into “a large quantity” and “a small number” groups; statistical analysis was performed using one-way ANOVA; *P* > 0.05 was considered statistically significant, with no significant correlation between the two variables

### Therapy

All 48 patients received supportive therapy. Among them, 35 patients (35/48) with severe ocular symptoms were treated with oral acyclovir tablets at a dose of 0.2 g five times a day for one week. Although most ocular surface infections are self-resolving, delayed treatment may exacerbate patients’ discomfort and even aggravate the condition, which is the reason for administering oral acyclovir to all patients with severe ocular symptoms. Additionally, two patients were treated with ganciclovir ophthalmic gel, instilled one drop four times a day, with all achieving symptom improvement within two weeks. Two other patients took ganciclovir capsules at a dose of 1 g three times a day, where one patient showed improvement within one month while the other had no improvement and suffered from disease recurrence. One patient received a combination therapy of acyclovir tablets, ganciclovir capsules and ganciclovir ophthalmic gel, resulting in symptom improvement within two weeks.

## Discussion

Infectious conjunctivitis is one of the most common acute ocular infections worldwide, characterized by high contagiousness. It affects approximately 2% of the global population, with viral conjunctivitis accounting for as high as 80% of all cases [17]. Due to the difficulty in distinguishing between viral and bacterial infections, clinicians often prescribe unnecessary antibiotics, thereby exacerbating the crisis of antimicrobial resistance [18]. Infectious keratitis is one of the leading causes of monocular blindness worldwide, particularly in developing countries. In China, it is a common blinding eye disease; approximately 32.37 million people are affected by corneal diseases, with a significant proportion attributed to infectious etiologies [19].

Epstein-Barr virus (EBV), a major γ-herpesvirus primarily transmitted via saliva, exhibits extremely high global prevalence, with approximately 90% of adults testing serologically positive; however, the majority of infected individuals remain asymptomatic and in a latent state [1]. However, when the immune homeostasis is disrupted, the latent EBV can be reactivated, leading to multisystem disorders involving the eye, including Acute Retinal Necrosis (ARN), uveitis, keratitis, and dry eye syndrome [5, 7, 8]. In certain regions (such as Vanuatu), EBV is detected as one of the viral causes of acute conjunctivitis, ranking second only to adenovirus [11].

Clinically, EBV-related ocular surface infections are often difficult to distinguish from conjunctivitis or keratitis caused by other pathogens due to their non-specific symptoms, leading to diagnostic challenges and treatment delays. Currently, research on EBV-infected ocular diseases lacks representative clinical feature analysis, and pathogen testing is time-consuming. In the early stages of illness, patients often require empirical treatment based on clinicians’ personal experience, making the diagnosis and management of EBV-infected ocular surface diseases extremely difficult. Understanding the risk factors for onset, common clinical features, and microbiological characteristics is crucial for ophthalmologists to handle these cases effectively. Therefore, in-depth analysis of its clinical features and etiological patterns is of vital importance for enhancing ophthalmologists’ understanding and diagnostic capabilities regarding this disease.

The results of this study indicate that patients with EBV-infected ocular surface diseases are predominantly young and middle-aged adults, with a male-to-female ratio of 1:1. This finding aligns with the widespread latent state of EBV in the adult population [1]. The identified risk factors for onset include a history of pet keeping, colds, allergies, travel history, foreign body exposure, previous ocular infections, chronic diseases, autoimmune disorders, and infectious diseases. EBV may induce ocular lesions either through direct infection or by indirectly affecting ocular tissues [7, 8], animals may harbor various bacteria and viruses; therefore, poor hygiene practices or excessive intimacy with pets can increase the likelihood of EBV infection in the ocular surface. The infection process of EBV involves latency and lytic replication, with the transition between these two states strictly regulated by the host immune status. Under specific conditions—such as immunosuppression, stress, or immunodeficiency—the virus can reactivate, leading to clinical symptoms [7, 20]. Common systemic conditions such as upper respiratory tract infections, allergic states, hyperglycemia, rheumatoid diseases, Sjögren’s syndrome, drug-induced keratitis, hepatitis B virus infection, and bacterial foreign body exposure can lead to immunological dysregulation. This compromised immune status facilitates the reactivation of latent EBV within the host. Furthermore, EBV-infected B and T cells promote the release of pro-inflammatory cytokines, including interleukin-10 (IL-10) and vascular endothelial growth factor (VEGF), thereby exacerbating ocular inflammation and angiogenesis [4, 7, 21]. This suggests that animals may act as viral carriers or indirectly promote infection by affecting the host’s immune environment, while colds and other stress states are key factors triggering the reactivation of latent viruses. This finding highlights the importance of inquiring in detail about patients’ pet contact history and recent health status during clinical history-taking for etiological tracing.

The majority of cases in this study presented with symptoms of foreign body sensation in the affected eye accompanied by yellowish discharge; tearing and blurred vision were also occasionally reported. Common signs included conjunctival hyperemia, follicles on the lower eyelid, papillae on the upper eyelid, and punctate epithelial defects on the cornea.Notably, most eyes exhibited yellow discharge, which differs from the typical clear watery discharge seen in viral conjunctivitis, suggesting either a mixed infection or a unique manifestation of EBV ocular infection. Analysis revealed that eyes with yellow discharge primarily exhibited mixed hyperemia, whereas those without yellow discharge showed predominantly simple conjunctival hyperemia.Although statistical analysis did not find a significant correlation between the presence of yellow discharge and neutrophils in the smear results, this may be attributed to the small sample size of this study, highlighting the need for further investigation with larger cohorts.It is worth noting that one patient developed a painless salmon-colored nodule on the left conjunctiva, consistent with the description reported by Alba Linero et al. [22]. However, unlike previous reports, this patient did not present with systemic infectious mononucleosis. Without performing a biopsy on the nodule, the patient received acyclovir antiviral therapy and supportive treatment for two months, after which the nodule gradually regressed and disappeared. A patient with rheumatoid disease presented with bilateral corneal ulcers, characterized by epithelial defects observed under slit-lamp microscopy. This clinical presentation is similar to the cases described by Yamashita, K., et al., highlighting the potential link between systemic autoimmune conditions and ocular EBV infection [7].

The majority of ocular surface scrapings exhibited a predominance of small round lymphocytes accompanied by reactive lymphocytes, often coexisting with a substantial number of neutrophils. A minority of samples revealed scattered eosinophils alongside heavy bacterial colonization; bacterial culture was positive in some cases. Furthermore, concurrent infections with adenovirus (ADV), herpes simplex virus (HSV), and microsporidia were identified in certain samples. Distinct clinical manifestations and management strategies were noted in ADV-EBV and HSV-EBV co-infections: ADV-EBV co-infections were characterized by SPKs without dendritic lesions and severe conjunctival inflammation, while HSV-EBV co-infections presented with typical corneal dendritic lesions plus severe ocular pain/photophobia. Corresponding management adjustments were made, including extended antiviral courses and avoided topical corticosteroids for HSV-EBV co-infections, which is consistent with clinical guidelines for HSV keratitis management. These findings indicate that immune dysregulation in patients with EBV-infected ocular surface diseases often leads to polymicrobial infections, highlighting the necessity for meticulous differential diagnosis and targeted antimicrobial therapy in clinical practice.

Ocular surface scraping can provide indirect supportive diagnostic evidence for viral ocular surface infection by rapidly identifying the inflammatory cell pattern associated with viral infection (lymphocyte-predominant infiltration) and ruling out bacterial/fungal infection, but this examination does not have diagnostic specificity for EBV infection—lymphocyte-predominant infiltration can also be seen in ocular surface infections caused by other viruses such as adenovirus and herpes simplex virus. Therefore, ocular surface scraping can only serve as an early auxiliary screening method for EBV-related ocular surface infection and cannot confirm EBV infection alone; a comprehensive diagnosis requires combination with ocular surface EBV-DNA quantitative PCR, serological antibody testing, and typical clinical symptoms and signs.

The study revealed significant differences in EBV viral load values between the symptomatic groups and among different follicle groups. Viral load plays a pivotal role in the pathogenesis and clinical outcomes of ocular infection. Notably, for the most common pathogen—human adenovirus (HAdV)—there exists a distinct “symptom threshold [23].” This study showed significant differences in EBV viral load among different symptom groups and different follicle groups, suggesting that viral load may be involved in the progression of EBV ocular surface infection. Although a “symptom threshold” for human adenovirus in ocular infection has been identified, there is no established literature supporting the quantitative association between EBV viral load and clinical symptoms, and whether a specific “symptom threshold” exists for EBV remains to be explored in more studies. Furthermore, our study observed the status of the fellow eyes; notably, some patients exhibited clinical symptoms in the fellow eye despite a positive qPCR result only in the affected eye, with symptoms in the fellow eye sometimes being more severe than those in the enrolled eye. This variability suggests that individual differences in immune status and concurrent infections likely contribute to the differential severity of ocular manifestations between eyes.

The diagnosis of EBV disease typically relies on demonstrating IgM heterophile antibodies during primary infection. These antibodies are detectable in up to 90% of adult cases12. IgM levels usually peak between the second and third weeks of acute infection and may remain positive for as long as one year34^2^. This test is simple, rapid, and cost-effective; however, it carries an inevitable false-negative rate of approximately 25% during the first week of illness. EBV-specific antibodies are present in 95% of adults and 50–85% of children. The detection of these specific antibodies—comprising antibodies against the viral capsid antigen (VCA), early antigen (EA), and nuclear antigen (EBNA)—facilitates the distinction between acute infection and past infection [15, 24]. In this study, EBV-specific antibody testing was performed on 30 patients. The results showed that all patients were negative for EBV IgM (early antigen) and EBV IgM (capsid antigen), while the majority of patients exhibited positive results for EBV IgG (capsid antigen) and EBV IgG (nuclear antigen). This indicates that most of the patients who visited the clinic had a history of previous EBV infection. The ocular manifestations in these patients are likely attributed to the reactivation of EBV under various conditions, such as immunosuppression, stress, or immune deficiency [7, 20]. As a result, this leads to ocular surface inflammation and lesions. Paying attention to the immune status of patients with ocular infections also aids in the diagnosis of EBV.

In clinical practice, it is crucial to clearly distinguish three states of EBV in ocular surface samples: primary EBV ocular surface infection, EBV reactivation-related ocular surface inflammation, and incidental EBV detection.

Primary EBV ocular surface infection is extremely rare in adults, characterized by positive EBV-specific IgM antibodies, accompanied by systemic manifestations such as fever, pharyngitis and lymphadenopathy, and a significantly elevated EBV-DNA load in local ocular surface samples. EBV reactivation-related ocular surface inflammation is the most common clinical form (such as all cases in this study), characterized by negative IgM antibodies, positive IgG antibodies, positive local EBV-DNA in the ocular surface, and the presence of immune dysfunction (cold, pet contact, autoimmune disease, etc.) as triggering factors. Incidental EBV detection is manifested as positive local EBV-DNA in the ocular surface, but no obvious ocular surface symptoms and signs, the patient’s immune status is normal, and the virus is only shed in ocular secretions without replication and pathogenicity.

The key to the differential diagnosis of the three lies in the combination of serological antibody profile, viral load level, clinical symptoms and signs, and host immune status assessment, so as to avoid misdiagnosing incidental viral detection as EBV infection or misjudging reactivation-related inflammation as primary infection.

EBV has an extremely high global seroprevalence, with approximately 90% of adults being seropositive and carrying latent virus for life after primary infection in childhood or adolescence. In this context, the positive detection of EBV-DNA in local ocular surface samples alone cannot directly confirm a causal relationship between EBV and ocular surface inflammatory lesions. The presence of local EBV-DNA may be a result of the spread of latent virus in the body to the ocular surface due to immune dysfunction, or even an incidental finding of viral shedding in the ocular secretions of asymptomatic carriers. Therefore, EBV-DNA positivity in local ocular surface samples is a necessary but not sufficient condition for diagnosing EBV-related ocular surface diseases, and the diagnosis can only be established by combining typical clinical symptoms and signs and excluding other etiologies.

To address the above limitations, this study adopted a comprehensive diagnostic strategy combining clinical symptoms, slit-lamp signs, local qPCR detection and serological antibody testing, rather than a single local EBV-DNA detection. All enrolled patients had typical ocular irritation symptoms and conjunctival hyperemia signs, with an extremely low detection rate of other common pathogenic bacteria (only 4 cases of Staphylococcus positive with low bacterial load), and some were co-infected with adenovirus/herpes simplex virus, excluding the possibility of ocular surface lesions caused by a single non-EBV pathogen. In addition, the complete negative of EBV-specific IgM antibodies ruled out acute primary infection, and the positive of IgG antibodies confirmed the history of latent EBV infection, further supporting that the local EBV-DNA positivity was not an incidental viral shedding, but the result of viral reactivation and replication in ocular surface tissues. This comprehensive diagnostic criterion effectively improved the diagnostic validity of EBV-related ocular surface inflammation and made the conclusion of the correlation between EBV and ocular surface lesions more reliable.

This study has several important limitations. Firstly, systematic detection of HHV-6, HHV-7 and HHV-8 was not conducted. Studies by Usui et al. have confirmed that HHV-6 and HHV-7 can be detected in ocular inflammatory diseases and are involved in the pathological damage process of the conjunctiva and cornea [25]. As members of the herpesvirus family like EBV, the above viruses share similar biological characteristics such as latency, immune-mediated reactivation and lymphoproliferation promotion. They may coexist with EBV and participate in the occurrence and development of ocular surface inflammation, and even may potentially interfere with the detection efficiency and result interpretation of EBV. Therefore, the EBV infection detected in this study is not the only pathogenic agent, and the possibility that HHV-6/7 undergo co-activation with EBV reactivation, or that undetected HHV-6/7 infection synergistically mediates ocular surface inflammatory damage with EBV cannot be excluded. Future studies need to establish a combined detection system for herpesviruses including EBV, HHV-6/7/8, clarify the co-infection pattern and interaction mechanism of these viruses in ocular surface infection, and provide more comprehensive experimental evidence for the etiological diagnosis of EBV-related ocular surface diseases. Secondly, no information regarding the contact history (e.g., exposure to EBV-infected individuals) or family history (e.g., familial aggregation of EBV-associated ocular surface infection) of the enrolled patients was collected and analyzed, which limits the further exploration of the transmission route and genetic susceptibility of EBV ocular surface infection. Furthermore, the patients’ immune status was not evaluated by direct laboratory assays such as lymphocyte subset counting or immunoglobulin level detection; immune dysfunction was only inferred indirectly through antibody profiles, clinical risk factors and manifestations, which limits the in-depth exploration of the correlation between immune status and EBV reactivation in ocular surface infection.

In addition, a standardized grading system was not used for the evaluation of lymphocytes in ocular surface scrapings in this study, and no interobserver consistency validation was performed. Subsequent studies can improve this evaluation system to enhance the objectivity and comparability of the results. Some subgroup analyses in this study had small sample sizes; although the statistical assumptions were met through normality and homogeneity of variance tests, the statistical power may be limited, and no correction was applied for multiple comparisons. The relevant results need to be further confirmed in confirmatory studies with larger sample sizes. The interpretation of marginal P-values is only a trend suggestion and does not have clear statistical causal significance. This study initially explored the association between EBV viral load and clinical symptoms and proposed a threshold speculation by analogy with adenovirus. This extrapolation is not supported by existing literature on EBV ocular surface infection and is only an exploratory observation, which needs to be further verified in subsequent studies with large sample sizes.

All patients received topical corticosteroids and antibiotics. Antiviral therapy was administered based on the severity of symptoms in the affected eye. Most patients experienced improvement in ocular symptoms within two weeks. Some authors have claimed that antiviral drugs should only be considered if the outcome is unsatisfactory following corticosteroid treatment [22]. The author suggests that waiting until corticosteroid therapy proves ineffective before adding antiviral agents may prolong the patient’s course of illness, increase discomfort, and potentially exacerbate the condition. Therefore, it is recommended to determine whether to initiate antiviral treatment based on the patient’s immune status, concurrent infections, and severity of ocular symptoms at the initial visit. The interpretation of marginal P-values in this study is only a hint of potential association trends without definite statistical causal significance, and the relevant conclusions need to be further verified by studies with larger sample sizes.

Ocular surface lesions associated with EBV in adults are mainly caused by the reactivation of latent virus (rather than primary infection), and such cases are prone to polymicrobial co-infections. A detailed medical history inquiry (especially for triggering factors such as pet contact and cold) and a comprehensive diagnostic evaluation (combining clinical symptoms, slit-lamp signs, local ocular surface EBV-DNA qPCR and serological antibody testing) are the keys to avoiding misdiagnosis and overdiagnosis. Ocular surface tissue scraping examination can rapidly and effectively provide indirect evidence for viral infection and assist in early differential diagnosis; local ocular surface EBV-DNA detection is a necessary but not sufficient condition for diagnosis, and the causal relationship between EBV and ocular surface lesions must be judged by comprehensive diagnostic criteria.

## Data Availability

No datasets were generated or analysed during the current study.
